# Machine learning-based radiomics approach assessing preoperative non-contrast CT for microsatellite instability prediction in colon cancer

**DOI:** 10.3389/fphys.2025.1672636

**Published:** 2025-09-29

**Authors:** Dongming Ren, Yingjuan Wang, Luda Chen, Jianfeng He, Tao Shen

**Affiliations:** 1 Faculty of Information Engineering and Automation, Kunming University of Science and Technology, Kunming, China; 2 Department of Radiology, The Third Affiliated Hospital of Kunming Medical University, Kunming, China; 3 Department of Colorectal Surgery, The Third Affiliated Hospital of Kunming Medical University, Kunming, China

**Keywords:** colon cancer, microsatellite instability, non-contrast CT, radiomics, machine learning

## Abstract

**Objectives:**

To assess the feasibility of non-contrast CT-based radiomics model for predicting microsatellite instability (MSI) status in colon cancer.

**Methods:**

Leveraging non-contrast abdominal CT imaging data from 57 retrospectively enrolled patients with balanced class distribution (training cohort: n = 38, 19 non-MSI-H and 19 MSI-H; test cohort: n = 19, 9 non-MSI-H and 10 MSI-H), we implemented a voxel volume-based tumor feature selection method. Feature selection integrated four feature selection filters—correlation analysis, univariate logistic regression, least absolute shrinkage and selection operator (LASSO), and recursive feature elimination (RFE). We comparatively evaluated multiple classifiers using cross-validation combined with accuracy for choosing the best classifier.

**Results:**

A multilayer perceptron-based classification model was developed, achieving average multifold accuracy of 0.871 in cross-validation on the training cohort. In the test cohort, the model achieved an AUC of 0.944 (95% CI 0.841–1.000) with accuracy of 0.842, while maintaining sensitivity of 0.889 and specificity of 0.800, demonstrating excellent and comparable performance to previous contrast-enhanced CT-based radiomics models.

**Conclusion:**

We validated the feasibility of non-contrast CT for MSI prediction in colon cancer with radiomics analysis, highlighting its potential as a flexible and cost-effective preliminary screening tool. This approach, which does not require supplementary medical examination, may enhance clinical decision-making by providing a valuable tool for identifying MSI-H molecular subtypes in colon cancer patients.

## 1 Introduction

Colon cancer ranks among the most prevalent malignancies globally, with epidemiological data documenting 1.14 million new cases and 530,000 associated deaths in 2022, reflecting a concerning upward trajectory in both incidence and mortality rates (Bray et al., 2024; Smith et al., 2002). Microsatellite instability has emerged as a pivotal molecular biomarker in colon oncology, with substantial evidence demonstrating differential therapeutic responses to immunotherapy and chemotherapy across the three MSI subcategories—Microsatellite Stable (MSS), Microsatellite Instability-Low (MSI-L), and Microsatellite Instability-High (MSI-H) (Zhao et al., 2019; Le et al., 2015). Notably, patients with MSI-H exhibit enhanced responsiveness to immune checkpoint inhibitors (ICIs) and more favorable prognostic (André et al., 2020), thereby demonstrating the value of MSI detection in guiding therapeutic strategies and prognostic evaluation for colon cancer management (Sinicrope and Sargent, 2012). Clinical practice guidelines recommend testing for MSI in all colon cancer patients (Benson et al., 2021).

Clinical methods predominantly used for MSI detection include polymerase chain reaction (PCR) and immunohistochemistry (IHC) (Söreide et al., 2006; Lindor et al., 2002). IHC evaluates mismatch repair (MMR) protein expression loss in tissue specimens and carries risks of false-negative interpretations, requiring 2–7 days for completion. While PCR remains the gold standard through direct microsatellite region length analysis (Umar et al., 2004), its implementation necessitates over a week of processing time and a higher cost. Both methods are expensive, depending on invasive biopsy sampling coupled with complex molecular techniques (Sepulveda et al., 2017; Harada and Morlote, 2020). The contradiction between the clinical demand for MSI testing and the high cost of conventional detection methods has created an urgent need for a non-invasive, low-cost approach that is desired by both patients and clinicians.

Radiomics-based feature analysis can capture the heterogeneous manifestations of tumors in medical imaging, providing a non-invasive and objective auxiliary tool for clinical diagnosis (Lambin et al., 2017). Existing studies have shown the feasibility of modeling using contrast-enhanced CT (CECT) imaging combined with radiomics to predict MSI status in colon cancer (Cao et al., 2021; Golia Pernicka et al., 2019; Bian et al., 2024; Li et al., 2021). Cao et al. integrated carcinoembryonic antigen (CEA), carbohydrate antigen 199 (CA199), and carbohydrate antigen 125 (CA125) with triphasic CECT for nomogram development (Cao et al., 2021). Golia et al. incorporated clinical information (e.g., lymph node positivity, mucinous adenocarcinoma status, KRAS mutation profiles) with venous-phase CECT in random forest modeling (Golia Pernicka et al., 2019). Bian et al. aggregated monophasic or multiphasic CECT with laboratory indices including CEA, CA199, white blood cell count, etc (Bian et al., 2024). Similarly, Li et al. incorporated venous-phase CT features with CEA and CA199 for machine learning model development (Li et al., 2021). These studies rely on multiphasic or venous-phase CECT and invasive auxiliary examinations to incorporate additional clinical features for enhancing the performance of the combination model. However, these supplementary tests not only increase prediction costs and procedural complexity but also diminish the non-invasive advantage of the method while amplifying data collection and integration challenges. These combined factors restrict the clinical implementation and widespread adoption of such models.

Notably, previous studies on the MSI status of colon cancer have paid insufficient attention to radiomic analysis of non-contrast CT (Wang et al., 2023). The use of non-contrast CT in tumor-related radiomics studies is common across various areas, including rectal cancer (Yuan et al., 2020), Hodgkin’s lymphoma (Jensen et al., 2022), adrenal tumors (Yuan et al., 2023), and gastrointestinal stromal tumors (Palatresi et al., 2022; Zhang et al., 2020; Xie et al., 2023), covering a wide range of tissue types. Yuan et al. conducted radiomics analysis on non-contrast CT scans of rectal cancer cases undergoing treatment to assess tumor regression grading (Yuan et al., 2020). Laura J. et al. also explored the feasibility of using non-contrast CT to evaluate the metabolic activity of Hodgkin’s lymphoma (Jensen et al., 2022). Additionally, researchers have attempted to use radiomic features extracted from non-contrast CT to differentiate between solitary micronodular adrenal hyperplasia and lipid-poor adenomas (Yuan et al., 2023). Non-contrast CT has also been used in multiple studies on gastrointestinal stromal tumors (Palatresi et al., 2022; Zhang et al., 2020; Xie et al., 2023). However, it remains unclear whether radiomics features extracted from non-contrast CT can be used to assess the MSI status of colon cancer currently.

Therefore, the aim of this study was to assess the feasibility of non-contrast CT-based radiomics model for predicting MSI status in colon cancer. We further validated this non-invasive approach, which integrates non-contrast CT imaging with routine clinical features to provide clinicians with a highly accessible and low-cost screening tool for detecting MSI status in colon cancer.

## 2 Materials and methods

### 2.1 Patient cohort

This study involving human participants was approved by the Medical Ethics Committee of the Third Affiliated Hospital of Kunming Medical University (Approval No. KYLX2023-198). All procedures were conducted in accordance with relevant guidelines and regulations, including the Declaration of Helsinki. As a retrospective study, informed consent was waived by the ethics committee.

The study cohort included patients with PCR-confirmed diagnoses (October 2020–January 2022). The collected data comprised preoperative CT scans, MSI status (non-MSI-H or MSI-H), and four clinical variables: age, sex, colon laterality (left/right), and subsite (ascending/transverse/descending/sigmoid). Feature selection prioritized readily accessible parameters, deliberately excluding histopathological, genomic, and even basic medical history data to minimize diagnostic complexity, clinician-patient communication, and resource utilization.

**FIGURE 1:**
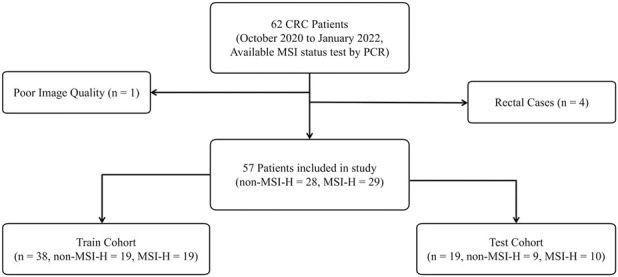
The inclusion and exclusion criteria for patients and the sample cohort were partitioned. Both the training and test cohorts exhibited a non-MSI-H to MSI-H class ratio of approximately 1:1, ensuring a balanced distribution of positive and negative classes.

Exclusion criteria comprised: 1) Rectal carcinomas based on distinct imaging and physiological characteristics compared to colonic malignancies (n = 4); 2) Suboptimal CT imaging quality per predefined criteria due to metal artifacts or motion (n = 1). The final cohort included 57 patients with colon cancer, stratified into training and test cohorts through randomized allocation with balanced classes, as detailed in Figure 1.

### 2.2 Assessment of MSI status

The PCR-based MSI detection protocol, considered the gold standard for MSI assessment in colon cancer, demonstrates high diagnostic accuracy with sensitivity and specificity ranges of 67%–100% and 61%–100%, respectively (Vilar and Gruber, 2010; Zhang and Li, 2013). Tumor and matched normal DNA samples were analyzed using fluorescence-labeled primers targeting five consensus microsatellite loci (BAT-25, BAT-26, D2S123, D5S346, D17S250) in our study, followed by capillary electrophoresis (Umar et al., 2004). MSI status was classified as MSI-H (≥ two unstable loci), MSI-L (single unstable locus), or MSS (no instability). All MSI assessments in this study were definitively determined by PCR, ensuring biologically validated labels for radiomics modeling.

### 2.3 CT imaging acquisition and segmentation

All imaging data were acquired using a standardized protocol on the same Siemens SOMATOM Definition AS+ 64-detector row CT scanner (128-slice configuration). Scanning parameters included: 120 kV tube voltage, automated tube current modulation via CareDose 4D technology, helical pitch 0.6, isotropic reconstruction at 2 mm slice thickness, and voxel spacing 0.8 × 0.8 × 2 mm (x, y, z). Anatomical coverage extended from 2 cm superior to the diaphragmatic dome to the inferior pubic symphysis, encompassing the entire abdominal cavity. All non-contrast imaging data were archived in DICOM format and retrieved through the institutional Picture Archiving and Communication System (PACS).

Region of interest (ROI) segmentation was conducted by one radiologist (15+ years’ experience) employing 3D Slicer (v5.6.2, https://www.slicer.org/) under blinded conditions to the pathology information of each patient. Following imaging quality verification, manual segmentation encompassed: primary tumor mass, adjacent bowel segments (5 mm margin), mesenteric fat infiltration zones, iliac vascular territories, and retroperitoneal nodal stations. The DICOM files with ROI were saved for subsequent radiomics analysis.

### 2.4 Feature extraction, selection, and model building

Preprocessing was implemented for non-contrast CT imaging comprising: 1) optimal windowing (width/level) configuration for abdominal soft tissue visualization; 2) intensity normalization (0–255 grayscale range). Preprocessed data were converted into integer discrete gray-level values with a bin width of 25 and then analyzed using PyRadiomics (version 3.1.0; http://www.radiomics.io/) (Van Griethuysen et al., 2017), an extensively used tool for quantitative imaging biomarker extraction. These biomarkers characterize tumor phenotypes, including intralesional heterogeneity patterns and spatial infiltration characteristics. For instance, the original_shape_VoxelVolume calculates three-dimensional tumor burden using the equation:

$$original\_shape\_VoxelVolume=\sum_{K=0}^{N}V_{k}$$

This biomarker is calculated by multiplying segmented voxel count by voxel-wise volume unit $V_{k}$ , equivalent to summing all tumor voxel volumes. Therefore, original_shape_VoxelVolume was used as the primary measure of tumor voxel in our study. Non-numeric radiomics features were removed after extraction. Each retained feature was Z-score standardized to reduce the scale differences between features. Since the MSI-H and non-MSI-H groups had similar sample sizes in the training cohort, no setting for class imbalance was applied in subsequent model development.

**FIGURE 2:**
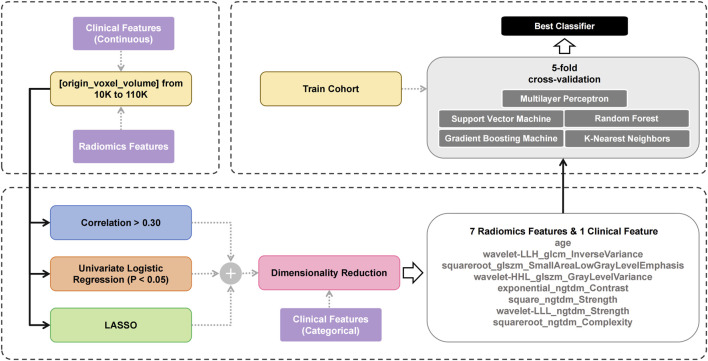
The workflow of feature selection and model building.

Previous studies have shown that the MSI status in colon cancer exhibits strong correlation with texture-related features (Cao et al., 2021; Wang et al., 2023). This study employed a systematic approach to identify texture-related and key features from non-contrast CT, with the selection process summarized in Figure 2. The workflow began by selecting tumors based on volumetric criteria (10,000 - 110,000 voxels). Features extracted from these selected tumors were then processed through three parallelized filtering methods with continuous clinical parameters. The filtering strategies comprised: 1) correlation coefficient analysis (absolute value of correlation coefficient is greater than 0.3), 2) univariate logistic regression (p < 0.05), and 3) LASSO (non-zero regression coefficient). Each filtering method independently screened the extracted features; in other words, a feature only needed to meet the criteria of one filter to pass the first-level screening. Then, the union of the results from the three methods was taken and subjected to a second-level screening using RFE to determine the optimal features. This second-level screening process also reduced the dimensionality and mitigated the risk of overfitting.

Five classical machine learning classifiers (support vector machines (SVM), gradient boosting decision trees (GBDT), k-nearest neighbors (KNN), random forest (RF), and multilayer perceptron (MLP)) were incorporated in this study. Each algorithm has strengths and limitations in handling data distributions, feature complexity, and noise (Caruana and Niculescu-Mizil, 2006; Fernández-Delgado et al., 2014), as shown in Table 1. Comparative evaluation facilitates the identification of classifiers that achieve an optimal balance between accuracy and computational efficiency, supporting model building that aligns with research requirements.

**TABLE 1:** The advantages and disadvantages of the five classifiers.

Classifier	Advantages	Disadvantages
SVM	- Handles high-dimensional data - Performs well with small sample sizes	- Long training time - Sensitive to parameter selection
GBDT	- Works well with diverse datasets - Captures non-linear relationships	- Prone to overfitting - High computational complexity - Sensitive to outliers
KNN	- No training required - Robust to outliers	- High prediction computational cost - Sensitive to the choice of K
Random Forest	- Balances accuracy and overfitting prevention - Good scalability	- May still overfit if the dataset is small - perform poorly on imbalanced datasets
MLP	- Versatile for classification and regression tasks - Can generalize well with small sample sizes if properly regularized	- Prone to getting stuck in local optima - Time-consuming training

Consequently, all classifiers were evaluated using the Stratified K-Fold algorithm with 5-fold cross-validation on the training cohort. During cross-validation, we used accuracy as a metric, combined with grid search, to determine the optimal hyperparameters for each classifier. The average accuracy across the five folds was used as the initial performance metric for the models. Subsequently, a more comprehensive comparison of the five classifiers was conducted on the test cohort using additional metrics to determine the final classifier to be used.

### 2.5 Statistical analysis

This study implemented a complete analytical workflow using Python (v3.8.19) with third-party libraries (scikit-learn v1.3.2, pandas v2.0.3) for data processing and model development. Clinical features were summarized as mean or interquartile range. Radiomics features were extracted via PyRadiomics. Feature selection combined correlation analysis (correlation value >0.30), LASSO, and univariate logistic regression (statistically significant differences P < 0.05). The dimensionality reduction was performed using RFE. The performance of each classifier was evaluated across the fivefold cross-validation with multifold mean accuracy. The final model performance was evaluated using accuracy, sensitivity, specificity, F1-score, precision, recall, and ROC analysis (AUC with 95% CI via DeLong test). This systematic approach ensured methodological rigor and reliable predictive performance.

## 3 Results

### 3.1 Patient profiles

This study enrolled 57 colon cancer patients (28 males, 29 females), divided into non-MSI-H and MSI-H groups. The MSI-H group was younger (50.7 vs. 61.8 years) and had a higher proportion of males. Most tumors were right-sided (71.9%), with the ascending colon as the primary subsite (57.9% overall; 58.6% in non-MSI-H). No significant differences were observed in subsite distribution (ascending/transverse/descending/sigmoid) or colon laterality (right/left) between groups, indicating similar spatial patterns. See Table 2 for complete clinical comparison.

**TABLE 2:** Patient profiles.

Cohort	All (n = 57)	Non-MSI-H (n = 29)	MSI-H (n = 28)
Age (Mean, IQR)	56.35 (48.0, 67.0)	61.83 (54.0, 69.0)	50.68 (44.8, 58.3)
Sex (n, %)			
Male	28 (49.1%)	11 (37.9%)	17 (60.7%)
Female	29 (50.9%)	18 (62.1%)	11 (39.3%)
Colon Laterality (n, %)			
Left Colon	16 (28.1%)	8 (27.6%)	8 (28.6%)
Right Colon	41 (71.9%)	21 (72.4%)	20 (71.4%)
Subsite (n, %)			
Descending Colon	10 (17.5%)	4 (13.8%)	6 (22.2%)
Sigmoid Colon	8 (14.0%)	6 (20.7%)	2 (7.4%)
Transverse Colon	6 (10.5%)	2 (6.9%)	4 (14.8%)
Ascending Colon	33 (57.9%)	17 (58.6%)	15 (55.6%)

### 3.2 Feature selection

**FIGURE 3:**
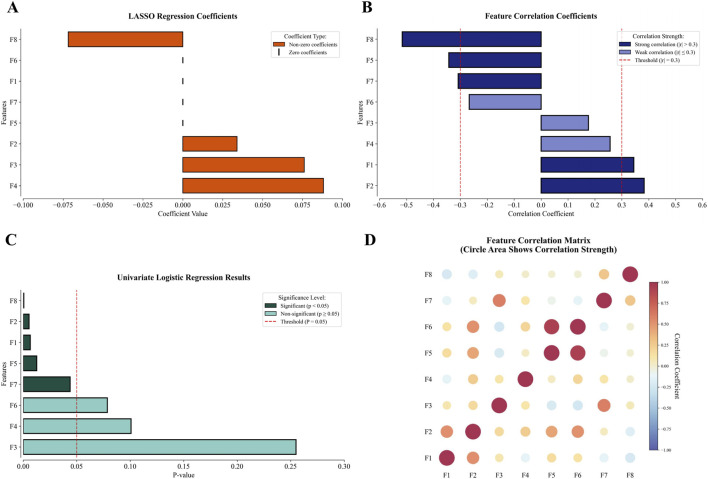
The performance of the 8 final modeling features in selection and correlation matrix (F1: wavelet-LLH_glcm_InverseVariance, F2: squareroot_glszm_SmallAreaLowGrayLevelEmphasis, F3: wavelet-HHL_glszm_GrayLevelVariance, F4: exponential_ngtdm_Contrast, F5: square_ngtdm_Strength, F6: wavelet-LLL_ngtdm_Strength, F7: squareroot_ngtdm_Complexity, F8: age). **(A)** In LASSO, four features showed non-zero coefficients, with the remaining features indicated by short vertical lines. **(B)** Correlation analysis revealed five features with strong correlations exceeding 0.3 and three with weaker correlations below 0.3. **(C)** Univariate logistic regression identified five statistically significant features, while three features remained non-significant. **(D)** The correlation between features.

These eight features exhibited variable prioritization across the three linear selection methodologies (Figure 3). Different screening methods have their own criteria. In LASSO, four features with non-zero regression coefficients are more important, while the remaining features are indicated by short vertical lines (Figure 3A). From the perspective of correlation analysis, five features are highly correlated with MSI-H (absolute value of the correlation coefficient exceeds 0.3), making them more important than the other three features (Figure 3B). Univariate logistic regression (Figure 3C) further emphasizes five statistically significant features (p < 0.05), indicating that these features are more predictive of MSI status. The correlations between the features are shown in Figure 3D.

The three linear methods—correlation analysis, univariate logistic regression, and LASSO—were applied to analyze 1,416 extracted features. The detailed results of these three linear filters are presented in the Supplementary Material. Each method identified distinct but interpretable associations with clinical outcomes. Univariate logistic regression selected 30 features, including 10 unique to this approach, by evaluating the individual contribution of each feature. LASSO chose 28 features with 19 method-exclusive identifiers, effectively controlling model complexity to prevent overfitting. Correlation analysis produced the most conservative results, retaining only 20 features. Implementing these methods in parallel leveraged their complementary strengths, enabling a comprehensive exploration of the feature space while reducing redundancy for improved model generalizability. The outputs from three linear filters were integrated with categorical clinical features, resulting in a pool of 54 features. Guided by established engineering standards to prevent overfitting—specifically applying the 20% dimensionality rule relative to the training cohort size (Guyon and Elisseeff, 2003) —and based on our training cohort size of 39, we performed RFE to reduce the feature pool to 8 relevant predictors: wavelet-LLH_glcm_InverseVariance, squareroot_glszm_SmallAreaLowGrayLevelEmphasis, wavelet-HHL_glszm_GrayLevelVariance, exponential_ngtdm_Contrast, square_ngtdm_Strength, wavelet-LLL_ngtdm_Strength, squareroot_ngtdm_Complexity, and age.

Traditional sequential screening may risk information loss through progressive elimination. The parallel integration of three selection techniques enabled synergistic feature evaluation while circumventing information attrition typical of serialized or single-method implementations. The resultant 8-feature set thus embodies complementary strengths from multiple selection paradigms. Notably, age emerged as the most discriminative parameter, demonstrating the highest performance in both correlation analysis (absolute coefficient: 0.516) and univariate logistic regression (P < 0.001), surpassing all radiomics features. This finding aligned with its significance in subsequent ablation studies, confirming its biological and clinical relevance.

### 3.3 Cross-validation

**FIGURE 4:**
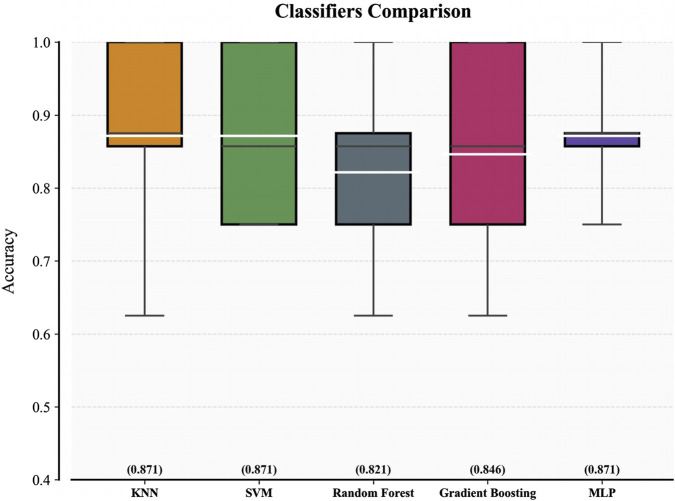
Comparison of multiple classifiers on the training cohort.

Figure 4 displays the five-fold cross-validation accuracy metrics for five classifiers trained on the 8-feature subset. The white line inside each box indicates the multifold mean accuracy (numerical values are displayed in brackets above the x-axis), and the black line represents the median. Three models—KNN, SVM, and MLP—demonstrated superior predictive capabilities with mean accuracy thresholds exceeding 0.85, indicating robust consistency in their high performance. Random Forest and Gradient Boosting underperformed slightly in contrast, with mean accuracies of 0.821 and 0.846, respectively. The fold-specific prediction accuracy of each classifier during 5-fold cross-validation is presented in Supplementary Material S2, revealing inter-fold variability that reflects the differential adaptability of models.

After determining the optimal hyperparameters for each classifier, we conducted a more comprehensive evaluation of the five classifiers and presented the results in Table 3. In terms of the AUC metric on the training set, SVM performed approximately 5% lower than MLP and KNN, leading to its exclusion. Although KNN had a 0.002 higher AUC than MLP, considering the size of the training set, we believe this slight difference is not significant enough to indicate that KNN has a decisive advantage over MLP, and further comparison is needed.

**TABLE 3:** Metrics of five classifiers on the training and test cohorts.

Cohort		MLP	KNN	SVM	Random forest	GBDT
Training	Accuracy	**0.871**	**0.871**	**0.871**	0.821	0.846
	AUC	0.895	**0.897**	0.841	0.808	0.841
	F1-score	**0.870**	**0.870**	**0.870**	0.817	0.842
	sensitivity	**0.800**	**0.800**	**0.800**	0.750	0.750
	specificity	**0.950**	**0.950**	**0.950**	0.900	**0.950**
Test	Accuracy	**0.842**	0.789	0.737	0.737	0.789
	AUC	**0.944**	0.867	0.722	0.811	0.811
	F1-score	**0.842**	0.789	0.737	0.737	0.789
	sensitivity	**0.889**	0.778	0.778	0.778	0.778
	specificity	**0.800**	**0.800**	0.700	0.700	**0.800**

The highest values for each metric are bolded in the Table 3. For the test, each classifier uses hyperparameters obtained based on accuracy during training, consistent with Figure 4.

Furthermore, according to the metrics on the test set, MLP demonstrated stronger generalization ability, showing more than a 5% advantage in accuracy, AUC, F1-score, and sensitivity. Considering multiple factors such as performance metrics, generalization ability, and runtime efficiency, MLP can meet the needs of real-world medical applications. Therefore, we chose MLP as the best classifier for the final test.

### 3.4 Performance of model

**FIGURE 5:**
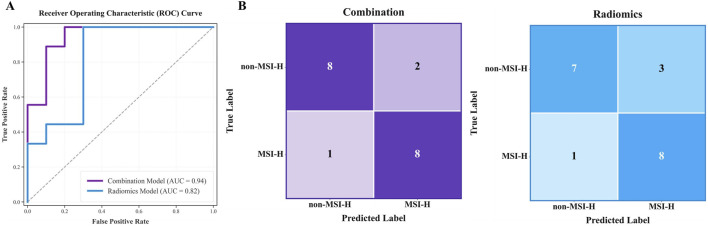
Test results for the combination model (including age) and radiomics model. **(A)** and **(B)** respectively display the AUC curves and confusion matrices of two models on the internal test cohort.

Two predictive models were developed using MLP classifier: the combination model (including age) with all eight features, and the radiomics model using seven radiomics features (excluding age). Both models were tested on the internal test cohort, with results presented in ROC curves (Figure 5A) and confusion matrices (Figure 5B). Two models were based on the same classifier and differed only in the inclusion of a single clinical feature (patient age); thus, these results also serve as an ablation experiment to target the impact of patient age.

The combination model performed better, with AUC 0.944 (95% CI 0.841–1.000) and accuracy 0.842, compared to AUC 0.822 and accuracy 0.789 for the radiomics model on the test cohort. Confusion matrix analysis showed the combined model made three errors with the higher specificity, while the radiomics model made four errors (misclassifying 3 Non-MSI-H cases). A more detailed performance comparison between the two prediction models is illustrated in Table 4: Apart from the sensitivity metric on the test cohort, the combination model outperforms the radiomics model in all other metrics: the combination model exhibits stronger discriminative power (an increase of 7.9% in AUC on the training cohort and 12.2% on the test cohort) and higher classification accuracy (an improvement of 5.0% in accuracy on the training cohort and 5.3% on the test cohort). These comparisons confirm that age significantly improves predictive reliability, aligning with the results of feature selection.

**TABLE 4:** Performance comparison between the combination model (including age) and the radiomics model.

Metrics	Radiomics		Combination	
	Training	Test	Training	Test
AUC	0.817	0.822	0.896	0.944
Accuracy	0.821	0.789	0.871	0.842
F1-score	0.797	0.788	0.854	0.842
Precision	0.893	0.805	0.960	0.847
Sensitivity	0.750	0.889	0.800	0.889
Specificity	0.900	0.700	0.950	0.800

## 4 Discussion

We developed a non-invasive method to predict MSI status (MSI-H vs. Non-MSI-H) using preoperative non-contrast CT scan and patient age in colon cancer. By analyzing tumor imaging features alongside routinely available clinical variables, this approach eliminates biopsy-related risks (e.g., infection, bleeding) and reduces human error in traditional tissue analysis. Age became the key clinical factor in the final model. The final model combining radiomics features and patient age achieved 84.2% accuracy and matching F1-score, proving our method worked reliably.

Our predictive method exhibits competitive potential compared with similar non-invasive studies utilizing CECT and eliminates the need for tissue biopsy, demonstrating its non-invasive nature. Cao et al. developed a radiomics model based on delayed-phase CECT, which achieved AUC of 0.953 and accuracy of 0.852 in the validation cohort (Cao et al., 2021). Ma et al. also developed a non-invasive predictive model using venous phase CECT, achieving AUC values of 0.903 and 0.852 in the training and test cohorts, respectively (Ma et al., 2024). The proposed non-contrast CT-based model achieved a multifold mean accuracy of 0.871 in the training cohort, with test cohort metrics including AUC 0.944, accuracy 0.842, sensitivity 0.889, and specificity 0.800. These results indicate comparable performance with CECT-based approaches while confirming the technical feasibility of non-contrast CT in predictive applications.

In the field of radiomics for oncology, the use of non-contrast CT has produced promising results. Yuan et al. developed and validated a radiomics nomogram to distinguish between solitary micronodular adrenal hyperplasia and lipid-poor adenomas, creating both a non-contrast radiomics model and a triphasic contrast-enhanced radiomics model. In an external cohort, there was no significant difference in AUC between the two models (0.838 vs. 0.843, p = 0.949) (Yuan et al., 2023). Although the clinical gold standard for diagnosing gastrointestinal stromal tumors is CECT (Palatresi et al., 2022), Zhang et al. demonstrated that radiomics models based on both non-contrast CT and CECT yielded similar AUC values for diagnosing high-malignant-potential gastrointestinal stromal tumors (Zhang et al., 2020). Similar findings were also reported by Xie et al. (2023) . These studies suggest that combining non-contrast CT with radiomics is a broadly feasible approach and, in some cases, non-contrast CT does not significantly underperform compared to CECT. For colon cancer, clinical practice tends to rely on CECT as the standard for diagnosis. We fully respect the views and standards of clinicians and do not think that non-contrast CT can replace CECT in clinical settings. However, for radiomics analysis alone, non-contrast CT may serve as an alternative option for feature extraction.

Previous radiomics research about MSI status in colon cancer thus lacks comparative validation between CECT and non-contrast CT, failing to disprove the feasibility of non-contrast CT for MSI prediction in colon cancer. Golia et al. first explored CT-based radiomics for predicting MSI status of colon cancer in 2019, choosing venous-phase CT for analysis based on standard care for TNM staging (Golia Pernicka et al., 2019). In 2021, Cao et al. speculated that the use of contrast agent could improve tissue differentiation through increased iodine concentration and consequently enhance prediction performance, though non-contrast CT was not discussed (Cao et al., 2021). A recent study by Ma et al. indicated their choice of venous-phase imaging for feature extraction was influenced by prior literature and their preliminary trial (Ma et al., 2023). Most similar studies either omit explanations for choosing CECT in radiomics feature analysis or briefly mention it as a limitation. CECT exposes patients to higher radiation doses with increased carcinogenic risks (Linet et al., 2012), while contrast agents carry nephrotoxicity and allergic reaction risks (Lameire et al., 2013), making it unsuitable for multiple reexaminations. From perspectives of cost, time consumption, and safety, non-contrast CT proves more advantageous for rapid screening and holds greater potential for implementation in resource-limited regions. Although non-contrast CT did not provide incremental diagnostic value in clinical practice, its widespread availability facilitates the radiomics quantitative analysis of colon cancer.

On the other hand, previous studies utilizing CECT have provided us with significant insights. Multiple studies have consistently identified texture-related features as predominant predictors (Cao et al., 2021; Golia Pernicka et al., 2019; Bian et al., 2024; Li et al., 2021; Wang et al., 2023), which play a crucial role in colon cancer MSI prediction by effectively reflecting tumor heterogeneity (Cao et al., 2021; Meng et al., 2019). Therefore, this study selected medium-to small-volume tumors (voxel counts ranging from 10,000 to 110,000) from our samples for feature selection. Within this voxel count range, the mean voxel count of MSI-H tumors was 47,857.570, slightly higher than the 43,680.635 observed in non-MSI-H tumors, thereby reducing volumetric discrepancies. Our method intentionally biases feature selection toward texture- or structure-related characteristics. Univariate logistic regression identified 10 first-order features (10/30) and 10 wavelet-related features (10/30). Correlation analysis revealed 6 first-order features (6/20) and 5 wavelet-related features (5/20). LASSO selected 5 first-order features (5/28) and 15 wavelet-related features (15/28). Our predictive model incorporated 8 features, with wavelet filters contributing 3 (3/8) and the remaining 5 presenting strong texture associations. The prominence of first-order category and wavelet-derived features in non-contrast CT feature analysis aligns with findings from multiple CECT studies (Cao et al., 2021; Golia Pernicka et al., 2019; Bian et al., 2024; Li et al., 2021), reinforcing the critical importance of texture features in MSI classification and showing the efficacy of our feature selection method for non-contrast CT imaging in highlighting the texture features of tumors.

Leveraging the practicality of non-contrast CT, the informatics system incorporating this model could serve as a rapid screening tool to facilitate clinical workflows. The workflow consists of three key steps: 1) standard non-contrast CT acquisition, 2) manual tumor segmentation requiring approximately 20 min, and 3) feature extraction by PyRadiomics and model prediction completed within 30 s. The system will deliver MSI predictions in 30 min—far faster and more cost-effective than traditional testing. The automated segmentation module, currently under development, is engineered to achieve comprehensive automation within 5 min after CT scan completion, integrating both image acquisition and computational analysis phases to support repeat screenings. Given the widespread use of non-contrast CT in routine health examination, this predictive method could enable large-scale screening of MSI status in colon cancer.

### 4.1 Limitations

This study has limitations that warrant attention. First, the rarity of MSI-H status in colon cancer constrained the positive sample size, resulting in a small-sample, single-center, retrospective design—a common challenge in similar research (Wang et al., 2023) — but internal consistency was maintained. Furthermore, the current lack of large-scale external datasets precludes comprehensive external validation. We acknowledge this work represents a preliminary exploration; future studies will expand the dataset through multicenter collaborations or ongoing data collection to enhance the robustness of conclusions and model generalization. Second, the cohort primarily comprised long-term residents from a single geographic region. Although current evidence on geographic influences on colon cancer MSI status remains limited and its clinical significance unclear, this homogeneity constrains generalizability to more diverse populations. Third, similar to other studies in this field, the features we have extracted and analyzed are influenced by the subjectivity of radiologists during manual segmentation. In the future, we plan to explore deep learning-based automatic segmentation methods to minimize bias in the segmentation process.

## 5 Conclusion

This study validated the feasibility of non-contrast CT in MSI assessment by radiomics machine learning modeling for colon cancer. The prediction model requires only patient age and non-contrast CT imaging as inputs, offering a non-invasive and simplified workflow with low implementation costs. This demonstrates its potential clinical utility as an efficient adjunct screening tool.

## Data Availability

The raw data supporting the conclusions of this article will be made available by the authors, without undue reservation.
