# Correction: Machine learning-based radiomics approach assessing preoperative non-contrast CT for microsatellite instability prediction in colon cancer

**DOI:** 10.3389/fphys.2026.1844252

**Published:** 2026-04-21

**Authors:** Dongming Ren, Yingjuan Wang, Luda Chen, Jianfeng He, Tao Shen

**Affiliations:** 1 Faculty of Information Engineering and Automation, Kunming University of Science and Technology, Kunming, China; 2 Department of Radiology, The Third Affiliated Hospital of Kunming Medical University, Kunming, China; 3 Department of Colorectal Surgery, The Third Affiliated Hospital of Kunming Medical University, Kunming, China

**Keywords:** colon cancer, microsatellite instability, non-contrast CT, radiomics, machine learning

An incorrect number was provided for The Yunnan Province Young and Middle-Aged Academic and Technical Leaders Project. The correct number is “202405AC350003”.

The original version of this article has been updated.

