# Supplementary material for: Machine learning-based radiomics approach assessing preoperative non-contrast CT for microsatellite instability prediction in colon cancer
Source: Front Physiol. 2025 Sep 29;16:1672636. doi: 10.3389/fphys.2025.1672636 (PMC12515810; doi:10.3389/fphys.2025.1672636)
Supplement: Supplementary file 1 [file Table1.docx]

Supplementary Material

# Supplementary Figures and Tables

## Supplementary Tables

**Supplementary Material S1.** **The screening results of the three filters.**

| **UnivariateLogisticRegression** | **Correlation** | **Lasso** |
| --- | --- | --- |
| age | age | age |
| original_firstorder_10Percentile | exponential_glcm_Imc1 | diagnostics_Image-original_Minimum |
| original_glszm_SmallAreaLowGrayLevelEmphasis | squareroot_glszm_SmallAreaLowGrayLevelEmphasis | original_shape_Sphericity |
| exponential_firstorder_10Percentile | logarithm_glszm_SmallAreaLowGrayLevelEmphasis | original_glszm_SmallAreaLowGrayLevelEmphasis |
| exponential_firstorder_Kurtosis | wavelet-LLH_glcm_InverseVariance | exponential_glcm_Imc1 |
| exponential_firstorder_Maximum | square_ngtdm_Strength | exponential_glcm_Imc2 |
| exponential_firstorder_Range | wavelet-HHL_firstorder_Mean | exponential_ngtdm_Contrast |
| exponential_firstorder_Skewness | square_glcm_ClusterShade | gradient_glcm_ClusterProminence |
| exponential_glcm_Imc1 | exponential_firstorder_Range | gradient_glszm_SmallAreaLowGrayLevelEmphasis |
| exponential_glcm_Imc2 | exponential_firstorder_Maximum | square_glcm_Idmn |
| exponential_glszm_GrayLevelVariance | original_glszm_SmallAreaLowGrayLevelEmphasis | squareroot_glszm_SmallAreaLowGrayLevelEmphasis |
| exponential_ngtdm_Strength | logarithm_firstorder_10Percentile | wavelet-LLH_firstorder_Mean |
| logarithm_firstorder_10Percentile | wavelet-LHH_glcm_Idmn | wavelet-LHL_firstorder_TotalEnergy |
| logarithm_glszm_SmallAreaLowGrayLevelEmphasis | wavelet-LHH_ngtdm_Contrast | wavelet-LHL_glszm_LargeAreaHighGrayLevelEmphasis |
| square_glcm_ClusterShade | exponential_glcm_Imc2 | wavelet-LHH_ngtdm_Contrast |
| square_ngtdm_Complexity | exponential_firstorder_Skewness | wavelet-LHH_ngtdm_Strength |
| square_ngtdm_Strength | wavelet-LHH_glcm_Idn | wavelet-HHL_firstorder_Mean |
| squareroot_firstorder_10Percentile | squareroot_ngtdm_Complexity | wavelet-HHL_firstorder_Skewness |
| squareroot_glszm_SmallAreaLowGrayLevelEmphasis | squareroot_firstorder_10Percentile | wavelet-HHL_glszm_GrayLevelVariance |
| squareroot_ngtdm_Complexity | square_ngtdm_Complexity | wavelet-HHH_glcm_Correlation |
| wavelet-LLH_glcm_InverseVariance |  | wavelet-HHH_gldm_DependenceEntropy |
| wavelet-LLH_glrlm_LongRunHighGrayLevelEmphasis |  | wavelet-HHH_gldm_DependenceNonUniformityNormalized |
| wavelet-LHH_glcm_Idmn |  | wavelet-HHH_glszm_LowGrayLevelZoneEmphasis |
| wavelet-LHH_glcm_Idn |  | wavelet-LLL_firstorder_Minimum |
| wavelet-LHH_ngtdm_Contrast |  | wavelet-LLL_glszm_ZoneVariance |
| wavelet-HLH_firstorder_Skewness |  | wavelet-LLL_ngtdm_Strength |
| wavelet-HLH_glcm_ClusterShade |  |  |
| wavelet-HHL_firstorder_Mean |  |  |
| wavelet-LLL_glcm_InverseVariance |  |  |
| wavelet-LLL_ngtdm_Complexity |  |  |

For the first-level feature selection (after Z-standardization and before RFE), the criteria for feature selection are P-value < 0.05 in univariate logistic regression, absolute value of correlation coefficient is greater than 0.30, or a non-zero LASSO regression coefficient (satisfying any one of these criteria is sufficient).

Supplementary Material S2 Prediction accuracy of various classifiers in each fold.

|  | **KNN** | **SVM** | **Random Forest** | **GBDT** | **MLP** |
| --- | --- | --- | --- | --- | --- |
| **Fold 1** | 1.000 | 1.000 | 0.875 | 1.000 | 0.875 |
| **Fold 2** | 0.625 | 0.750 | 0.625 | 0.625 | 0.750 |
| **Fold 3** | 0.875 | 0.750 | 0.750 | 0.750 | 0.875 |
| **Fold 4** | 0.857 | 0.857 | 0.857 | 0.857 | 0.857 |
| **Fold 5** | 1.000 | 1.000 | 1.000 | 1.000 | 1.000 |
